# Validation of a translated version of the modified Japanese Orthopedic Association (mJOA) cervical myelopathy score in an Arabic speaking population

**DOI:** 10.1051/sicotj/2021043

**Published:** 2021-09-14

**Authors:** Belal Elnady, Ahmed Abdelazim A. Hassan, Khaled Mohamed Hassan, Hassan Mohamed Ali

**Affiliations:** 1 Department of Orthopedics and Trauma Surgery, Assiut University Hospitals 71515 Assiut Egypt

**Keywords:** mJOA, Arabic translation, Validation, Degenerative cervical myelopathy, Psychometric analysis

## Abstract

*Introduction*: Degenerative Cervical Myelopathy (DCM) is a growing disorder. Standardization of its assessment tools is an integral part of its management. The modified Japanese orthopedic association (mJOA) score is one of the most commonly used tools. Currently, there is no available Arabic translated version of any cervical myelopathy functional score. This study aimed to translate, culturally adapt, and measure the psychometric properties of an Arabic translated version of the mJOA. *Methods*: After translation of the score using the standard forward-backward translation procedure, a validation study including 100 patients was carried out from June 2019 to June 2020. The following psychometric properties were measured: feasibility, reliability, internal consistency, validity, minimal clinically important difference (MCID), ceiling, and floor effect. *Results*: No problems were encountered during the process of translation and cross-cultural adaptation of the score. The mJOA-AR was found to be a feasible score. It showed high inter-observer reliability (*r* = 0.833, *P* < 0.001), test-retest reliability (*r* = 0.987, *P* < 0.001) and good internal consistency using Cronbach’s alpha (0.777) and Pearson interclass correlation coefficient (*r* = 0.717). The score showed good convergent and divergent construct validity correlating it to the Arabic validated version of the neck disability index (NDI). The mJOA-AR had an MCID of 1.506. Both the ceiling and floor effects of the total score and the first and second domains were within the acceptable range, while the third and fourth domains had a high ceiling effect (30% and 39%, respectively). *Discussion*: Our translated version of the mJOA score was found to be a feasible score with acceptable psychometric properties. This score can be utilized as a good outcome measure tool in Arabic-speaking countries.

## Introduction

Degenerative cervical myelopathy (DCM) is the leading cause of spinal cord dysfunction with increasing incidence in countries with an aging population. DCM is an incapacitating disease that hinders individuals from performing their simple daily activities and greatly affects their quality of life. It is also considered a burden from both the economic and social points of view [1, 2].

With the growing disease burden, objective assessment of patients with DCM became a pivotal point in managing the disease. The modified Japanese Orthopedic Association (mJOA) score is considered one of the most accepted and widely used scores to assess the functional status of patients [3]. The mJOA has been well studied in people suffering from DCM and is considered one of the predictors of the outcome after surgical intervention [3–5].

The mJOA has been translated and validated into Italian, Brazilian Portuguese, Dutch and Persian paving the way for other translated versions [2, 6–8]. Providing a translated version of a specific score helps ensure equivalence to the original score and reduces bias in the study [9]. A translated version of any cervical myelopathy functional scores has not been studied within an Arabic-speaking population, limiting the ability to objectively assess DCM and share validated outcomes from a cohort of nearly 400 million individuals which necessitates performing validation studies for the most commonly used scores. This study aimed to translate, culturally adapt and measure the psychometric properties of an Arabic translated version of the mJOA to justify using this score in Arabic-speaking countries.

## Materials and methods

Between June 2019 and June 2020, one hundred patients with DCM were recruited from the spine outpatient clinic. Patients older than 18 years with DCM clinical and radiological manifestations were included while patients with previous surgery were excluded.

The sample size was determined using power analysis (considering an alpha error of 0.05 and a power of 90%). A minimum sample size of 92 patients was needed for strong correlation, which was increased to 100 patients [10].

### Translation and cross-cultural adaptation

The process of forward-backward translation with independent translations and counter-translation was performed using the guidelines set by Guillemin et al. [11]. The following steps were performed:

1. The original mJOA Score was forward translated into Arabic by two independent professional translators (one mother tongue Arabic, fluent in English, and one mother tongue English fluent in Arabic).
2. These translations were reviewed, and the first draft was developed.
3. The first draft was back-translated into English by two professional English translators.
4. The forward and back translations were reviewed, and the second version was developed by an expert linguistic translator specialized in medical questionnaires.

After reaching the final version, the cognitive assessment was performed on ten patients to check for any linguistic or verbal difficulties.

### Psychometric properties analysis

For measuring the psychometric properties, each patient performed two visits. In the first visit, the score was applied to the patients by two different physicians, then a second visit was performed 14 days later to assess the test-retest reliability.

#### Feasibility

The time for score completion was calculated. Also, all the data was checked for missing or multiple responses.

#### Reliability

A fourteen-day test-retest reliability was applied. Its results were examined by using the Spearman test for the whole questionnaire and each domain independently. Inter-observer reliability was analyzed using the Kappa statistic to determine the consistency among the two observers for the whole questionnaire and each domain.

#### Internal consistency

Cronbach’s alpha was used to measure internal consistency for the whole questionnaire and after removing one domain at a time. Internal consistency was also measured by using the Pearson inter-item correlation coefficient.

### Validity

Both convergent and divergent construct validity was measured. Convergent construct validity was measured by correlating the mJOA-AR, and its domains with the results of the Arabic validated version of the Neck disability index (NDI) [12] using Pearson’s correlation, while divergent construct validity was measured by correlating the total mJOA-Ar score with items of the NDI that were expected to differ from the mJOA-Ar (reading, headache, concentration, and sleep). The NDI was used being the only available neck functional disability index that has been translated and validated into Arabic.

### Minimum Clinically Important Difference (MCID)

It was determined using a distribution-based method. Norman et al. proposed the standard deviation method and reported that, in patients with chronic disease, the MCID equals half a standard deviation of baseline scores [13].

#### Ceiling and floor effect

It is defined as the proportion of individuals that have scored either the highest (ceiling) or lowest (floor) possible score. They were calculated for the whole score and each domain.

### Data entry and statistical analysis

Data entry was performed using Microsoft Excel^®^, and the statistical analysis was done using the IBM SPSS^®^ version 27 [14, 15].

This study was reviewed and accepted by the Institutional Review Board (IRB) of our institution, and informed written consent was obtained from the patients before enrollment in the study.

## Results

### Demographic data (Table 1)

Out of the 100 patients involved in our study, 63 were males, and 37 were females. The patient’s age ranged from 29 to 77 years with a mean age of 50.6 ± 12.9 years.

**Table 1:** Demographic Data of the enrolled patients.

	Number (*n* = 100)	Percent (%)
Sex		
Male	63	63
Female	37	37
Residence		
Urban	46	46
Rural	54	54
Employment:		
Employed	63	63
Manual worker	32	50.8
Blue-collar worker	31	49.2
Unemployed	37	37

### Translation and cross-cultural adaptation

No major problems were observed during forward and back translation of the score with linguistic or grammatical errors. The only expression that was replaced by an equivalent Arabic synonym was in the motor dysfunction of the lower limb domain, in which the term “smooth reciprocation” was replaced by “walk with alternating footsteps”.

### Feasibility

The score was filled without any major difficulties (mean time required: 4 min, range 3–7 min). None of the investigators or patients reported an inability to complete the score because of linguistic or perceptive problems, also there were no missing or multiple answers.

### Reliability

The score showed good inter-observer and test-retest reliability. The inter-observer reliability using the Kappa statistic was outstanding for the total score (*r* = 0.833, *P* < 0.001) and for each domain (*r* = 0.889, *r* = 0.926, *r* = 0.939 and *r* = 0.984, *P* < 0.001 respectively).The test-retest reliability using the Spearman’s correlation showed strong correlations for the total score (*r* = 0.987, *P* < 0.001) and for each domain (*r* = 0.979, *r* = 0.98, *r* = 0.912 and *r* = 0.971, *P* < 0.001), respectively.

#### Internal consistency

The total score had high internal consistency. Using Cronbach’s alpha, it was found to be 0.777, and after removal of each domain was found to be 0.740, 0.684, 0.770, and 0.761, respectively. The largest drop was observed after removing the MDLE (motor dysfunction of the lower extremity) domain which dropped to 0.684. Internal consistency was also estimated by Pearson inter-item correlation coefficient (ICC). The total mJOA-Ar score was highly correlated with both the MDUE (motor dysfunction of the upper extremity) and MDLE domains (*r* = 0.717 and *r* = 0.826, respectively) and moderately correlated with the SDUE (sensory dysfunction of the upper extremity) and SD (sphincter dysfunction) domains (*r* = 0.619 and *r* = 0.691, respectively). Regarding the individual domains, MDUE was moderately correlated with all the other domains (*r* = 0.397, *r* = 0.395, and *r* = 0.318, respectively), The MDLE was weakly correlated with the SDUE domain (*r* = 0.224) and moderately correlated with the SD domain (*r* = 0.462), and the SDUE domain score was moderately correlated with the SD domain (*r* = 0.389) (Tables 2 and 3).

**Table 2:** Cronbach’s alpha for the whole score and after removing each domain at a time.

	No item excluded	MDUE	MDLE	SDUE	SD
Chronbach’s alpha	0.777	0.740	0.684	0.770	0.761

**Table 3:** Pearson inter-item correlation coefficient.

	MDUE	MDLE	SDUE	SD
Total score	0.717	0.826	0.619	0.691
MDUE		0.397	0.395	0.318
MDLE			0.224	0.462
SDUE				0.389

#### Validity

Regarding convergent construct validity, the total score was strongly correlated with the Arabic NDI (*r* = −0.876, *P* < 0.001) while the MDUL, MDLE, SDUE, and SD domains were moderately correlated (*r* = −0.647, *r* = −0.678, *r* = −0.490 and *r* = 0.537, *P* < 0.001, respectively). On the other hand, regarding divergent construct validity, the total mJOA-Ar score was found to be weakly correlated to the items of the NDI (*r* = −0.177, *r* = −0.220, *r* = −0.258 and *r* = −0.193, *P* < 0.001, respectively).

#### Minimum Clinically Important Difference (MCID):

It was found to be 1.506 (based on a standard deviation of 3.012).

#### Ceiling and floor effect

The ceiling and floor effect of the total score and the first two domains were acceptable, unlike those for the third and fourth domains. The total mJOA-Ar score had a ceiling and floor effect of 0%. Both the MDUE domain and the MDLE domain had a ceiling effect of 6% and a floor effect of 0%, the SDUE domain had a ceiling effect of 30% and a floor effect of 1%, and the SD domain had a ceiling effect of 39% and a floor effect of 0%.

## Discussion

Recently, research into the field of cross-cultural adaptation and psychometric properties analysis of patient-reported outcome measures in the field of orthopedics and spine surgery has gained much momentum, especially in Arabic speaking countries in the past few years, leading to the emergence of many Arabic translated and validated scores [16, 17]. DCM is a growing disease with increasing global concern. Research in all aspects of its management is increasing exponentially over the last two decades. One of the cornerstones of this research effort is identifying the appropriate tools and outcome measures for assessing and monitoring the disease [18, 19]. Overall, the psychometric properties of our translated version were acceptable, and no problems we encountered regarding the acceptability of the score or its comprehension by the patients.

The main limitation of this study was the absence of an intervention. This prevented us from measuring the responsiveness of the score and its sensitivity to change. It also prevented us from calculating the MCID by using anchor-based methods.

During the process of forward and back translation of the original mJOA score into Arabic, the only expression that had to be changed was in the MDLE domain to be properly understood within an Arabic dialect. The same expression was changed in both the Italian and Brazilian Portuguese translations of the score [2, 5].

Regarding its feasibility, the score had no missing or multiple answers. It was completed in a relatively short period of time compared to the time required to complete the Italian version [2].

Our study demonstrated high inter-observer and test-retest reliability of the mJOA-Ar and each of its domains, showing an outstanding Kappa statistic and a strong Spearman’s correlation, respectively. These findings are consistent with those of the English mJOA score and its Italian and Persian versions [2, 8, 20]. On the other hand, none of the previously mentioned studies calculated the Kappa coefficient for each domain.

The internal consistency of our mJOA-Ar established a strong correlation for the whole test and after removal of each domain, respectively, using Cronbach’s alpha. The largest drop was observed after the removal of the MDLE domain. Both Kopjar et al. and the Italian version reported a modest correlation for the mJOA score with a significant drop after removal of the MDUE domain [2, 3], while the Persian version showed similar results to our study with a higher Cronbach’s alpha and no significant drop after removal of any domain [8]. Using Pearson inter-item correlation coefficient, the internal consistency of the total mJOA-Arabic score was highly correlated with both MDUE and moderately correlated with MDLE, SDUE, and SD. With respect to individual scale components, MDUE was moderately correlated with MDLE and SDUE. The SD score was weakly associated with the SDUE components of the mJOA-Arabic, which is similar to both the English and Italian mJOA [2, 3].

Our score demonstrated both convergent and divergent construct validity by correlating it to the Arabic NDI and its individual domains, which contradicts the findings in other studies that demonstrated a weak correlation between the mJOA and the NDI [2, 3], unlike in the Brazilian Portuguese version where it was strongly correlated with the NDI [6] (Table 4).

**Table 4:** Results of the psychometric analysis performed on different translated versions of the mJOA scores.

	Feasibility (Completion time)	Test-retest reliability	Inter-observer reliability	Internal consistency*	Convergent construct validity**	MCID	Ceiling and floor effect
Italian version [2]	3–10 min	0.910	0.800	0.600	−0.330	NR	NR
Brazilian Portuguese version [6]	NR	NR	NR	NR	−0.752	NR	NR
Dutch version [7]	NR	NR	0.780	NR	NR	NR	NR
Persian version [8]	NR	NR	0.750	0.770	NR	NR	NR

NR = Not Reported.

* Using Cronbach’s alpha for the total score.

** By correlating it to a translated version of the Neck disability Index (NDI).

These differences in the results of both internal consistency and construct validity between different studies can be explained by the presence of multiple other factors that can affect both the quality of life and the patient-reported outcomes in patients with DCM which requires the use of multiple assessment tools together to properly assess patients with DCM. No single score or scale is considered a gold standard in the management of DCM, and this fact was supported in other multiple studies [2, 3, 18, 19].

Regarding the MCID, it was consistent with the findings in the study done by Tetreault et al. [21]. The main difference was that in our study, the MCID was calculated using only distribution-based methods, while in the other study it was calculated using three different methods: distribution-based, anchor-based, and the Delphi method.

The ceiling and floor effect of the mJOA has not been reported before. Both the ceiling and floor effects of the total score and the MDLE and MDUE were within the desired range while on the other hand, the SDUE and SD domains had a high ceiling effect which reflects the inability of these two domains to discriminate individuals at the higher end of the scale which may represent a problem on trying to identify the change in these domains. This may be attributed to the limited number of possible answers in these two domains compared to the other ones.

This is the first Arabic translation and validation study of the original English version of mJOA score to the best of our knowledge. It should be used in the clinical and research settings of Arabic-speaking countries to objectively evaluate patients, report outcomes, and compare them with international literature.

## Conclusion

Our translated version of the mJOA score was found to be both a feasible and reliable instrument for assessing people suffering from DCM with good psychometric properties. This score can be utilized as a good outcome tool for use in Arabic-speaking countries.

## Conflict of interest

BE certifies that he or she has no financial conflict of interest (e.g., consultancies, stock ownership, equity interest, patent/licensing arrangements, etc.) in connection with this article.

AH certifies that he or she has no financial conflict of interest (e.g., consultancies, stock ownership, equity interest, patent/licensing arrangements, etc.) in connection with this article.

KH certifies that he or she has no financial conflict of interest (e.g., consultancies, stock ownership, equity interest, patent/licensing arrangements, etc.) in connection with this article.

HA certifies that he or she has no financial conflict of interest (e.g., consultancies, stock ownership, equity interest, patent/licensing arrangements, etc.) in connection with this article.

## Funding

This research did not receive any specific funding.

## Ethics approval

The study has been reviewed and approved by the IRB of our institute (IRB no. 17100783).

## Informed consent

Written informed consent was obtained from all patients and/or families.

## Authors’ contributions

All authors contributed to the study’s conception and design. Material preparation, data collection, and analysis were performed by A. Hassan. B. Elnady wrote the first draft of the manuscript and all authors commented on previous versions of the manuscript. All authors read and approved the final manuscript.

## References

[1] Wilson JRF, Badhiwala JH, Moghaddamjou A, Martin AR, Fehlings MG (2019) Degenerative cervical myelopathy; A review of the latest advances and future directions in management. Neurospine 16(3), 494–505. 31476852 10.14245/ns.1938314.157 PMC6790745

[2] Longo UG, Berton A, Denaro L, Salvatore G, Denaro V (2016) Development of the Italian version of the modified Japanese orthopaedic association score (mJOA-IT): cross-cultural adaptation, reliability, validity and responsiveness. Eur Spine J 25(9), 2952–2957. 26961972 10.1007/s00586-016-4512-6

[3] Kopjar B, Tetreault L, Kalsi-Ryan S, Fehlings M (2015) Psychometric properties of the modified Japanese Orthopaedic Association scale in patients with cervical spondylotic myelopathy. Spine (Phila Pa 1976) 40(1), E23–E28. 25341993 10.1097/BRS.0000000000000648

[4] Kato S, Oshima Y, Oka H, et al. (2015) Comparison of the Japanese Orthopaedic Association (JOA) score and modified JOA (mJOA) score for the assessment of cervical myelopathy: a multicenter observational study [published correction appears in PLoS One. 2015;10(5):e0128392] PLoS One 10(4), e0123022. 25837285 10.1371/journal.pone.0123022 PMC4383381

[5] Tetreault LA, Côté P, Kopjar B, Arnold P, Fehlings MG (2015) AOSpine North America and International Clinical Trial Research Network. A clinical prediction model to assess surgical outcome in patients with cervical spondylotic myelopathy: internal and external validations using the prospective multicenter AOSpine North American and international datasets of 743 patients. Spine J 15(3), 388–397. 25549860 10.1016/j.spinee.2014.12.145

[6] Augusto MT, Diniz JM, Dantas FLR, Oliveira MF, Rotta JM, Botelho RV (2018) Development of the Portuguese version of the modified Japanese orthopaedic association score: cross-cultural adaptation, reliability, validity, and responsiveness. World Neurosurg 116, e1092–e1097. 29864576 10.1016/j.wneu.2018.05.173

[7] Bartels RH, Verbeek AL, Benzel EC, Fehlings MG, Guiot BH (2010) Validation of a translated version of the modified Japanese orthopaedic association score to assess outcomes in cervical spondylotic myelopathy: an approach to globalize outcomes assessment tools. Neurosurgery 66(5), 1013–1016. 20404709 10.1227/01.NEU.0000368391.79314.6F

[8] Azimi P, Shahzadi S, Benzel EC, Montazari A (2012) Functional evaluation using the modified Japanese Orthopedic Association score (mJOA) for cervical spondylotic myelopathy disease by age, gender, and type of disease. J Inj Violence Res 4(3 Suppl 1), 42.21502787

[9] Herdman M, Fox-Rushby J, Badia X (1998) A model of equivalence in the cultural adaptation of HRQoL instruments: the universalist approach. *Qual Life Res* 7(4), 323–335. 9610216 10.1023/a:1024985930536

[10] Tomczak M, Tomczak E, Kleka P, Lew R (2014) Using power analysis to estimate appropriate sample size. Trends Sport Sci 21(4), 195–206.

[11] Guillemin F, Bombardier C, Beaton D (1993) Cross-cultural adaptation of health-related quality of life measures: literature review and proposed guidelines. J Clin Epidemiol 46(12), 1417–1432. 8263569 10.1016/0895-4356(93)90142-n

[12] Shaheen AA, Omar MT, Vernon H (2013) Cross-cultural adaptation, reliability, and validity of the Arabic version of neck disability index in patients with neck pain. Spine (Phila Pa 1976) 38(10), E609–E615. 23429690 10.1097/BRS.0b013e31828b2d09

[13] Norman GR, Sloan JA, Wyrwich KW (2003) Interpretation of changes in health-related quality of life: the remarkable universality of half a standard deviation. Med Care 41(5), 582–592. 12719681 10.1097/01.MLR.0000062554.74615.4C

[14] Microsoft Corporation (2018). Microsoft Excel. Retrieved from https://office.microsoft.com/excel.

[15] IBM Corp. Released (2020) IBM SPSS Statistics for Windows, Version 27.0. IBM Corp, Armonk, NY.

[16] Ahmed KM, Said HG, Ramadan EKA, Abd El-Radi M, El-Assal MA (2019) Arabic translation and validation of three knee scores, Lysholm Knee Score (LKS), Oxford Knee Score (OKS), and International Knee Documentation Committee Subjective Knee Form (IKDC) [published correction appears in SICOT J. 2019;5:27]. SICOT J. 5, 6. 30848244 10.1051/sicotj/2018054 PMC6407430

[17] Hanbali Y, Perry T, Hanif A, et al. (2019) Reliability and validity of the Arabic version of the Early Onset Scoliosis 24 Items Questionnaire (EOSQ-24). Sicot J 205, 7. 10.1051/sicotj/2019001 PMC6405253 30834888

[18] Kalsi-Ryan S, Singh A, Massicotte EM, et al. (2013) Ancillary outcome measures for assessment of individuals with cervical spondylotic myelopathy. Spine (Phila Pa 1976) 38(22 Suppl 1), S111–S122. 23963009 10.1097/BRS.0b013e3182a7f499

[19] Singh A, Tetreault L, Casey A, Laing R, Statham P, Fehlings MG (2015) A summary of assessment tools for patients suffering from cervical spondylotic myelopathy: a systematic review on validity, reliability and responsiveness. Eur Spine J 24(Suppl 2), 209–228. 10.1007/s00586-013-2935-x 24005994

[20] Yonenobu K, Abumi K, Nagata K, Taketomi E, Ueyama K (2001) Interobserver and intraobserver reliability of the Japanese orthopaedic association scoring system for evaluation of cervical compression myelopathy. Spine (Phila Pa 1976) 26(17), 1890–1895. 11568701 10.1097/00007632-200109010-00014

[21] Tetreault L, Nouri A, Kopjar B, Côté P, Fehlings MG (2015) The minimum clinically important difference of the modified Japanese orthopaedic association scale in patients with degenerative cervical myelopathy. Spine (Phila Pa 1976) 40(21), 1653–1659. 26502097 10.1097/BRS.0000000000001127

